# Small RNA-mediated suppression of sex chromosome meiotic conflicts during *Drosophila* male gametogenesis

**DOI:** 10.1042/BST20240344

**Published:** 2025-02-06

**Authors:** Jeffrey Vedanayagam

**Affiliations:** Department of Neuroscience, Developmental and Regenerative Biology, University of Texas at San Antonio, San Antonio, TX 78249

**Keywords:** arms race, drosophila, meiotic drive, piRNA pathway, RNA interference

## Abstract

Meiosis is an evolutionarily conserved process in eukaryotes that ensures equal segregation of alleles and chromosomes during reproduction. Although parity in allelic transmission is the norm, selfish genes such as meiotic drivers can violate Mendel’s first law of segregation. Sex chromosome drive is a form of meiotic drive that leads to unequal segregation of sex chromosomes, resulting in sex-ratio distortion and/or sterility in the offspring. Adverse fitness effects due to sex chromosome drive trigger the evolution of suppressors to restore Mendelian segregation. However, the molecular mechanisms by which suppressors emerge and counteract meiotic drive genes remain unclear. Recent studies from *Drosophila* have shed light on the critical roles of small RNA-mediated post-transcriptional silencing in mitigating sex chromosome meiotic conflicts. This review highlights the recruitment of two distinct small RNA pathways to combat intragenomic conflicts during male gametogenesis and seeks to reveal the impact of molecular arms races between meiotic drivers and their suppressors in shaping genome and sex chromosome evolution.

## Introduction

In sexually reproducing organisms, meiosis is a process by which haploid gametes (e.g. egg and sperm cells) are produced from diploid parental cells [1]. One of the hallmarks of meiosis is the equal segregation of genes/alleles [2]. Meiotic drive is a form of genetic cheating that violates this norm, resulting in skewed transmission of alleles [3]. Indeed, this phenomenon is widespread and has been reported in yeasts [4], many different arthropods [5,6], plants [7], mice [8,9], and at least one instance in humans [10]. The extent to which such violations of Mendelian segregation occur in different organisms remains unknown, as meiotic drive may be unrecognized in the absence of overt phenotypes; however, sex chromosome meiotic drive can readily be observed as a skewed male-to-female ratio among progeny [5]. Although sex chromosome drive has been documented in numerous species, the genes responsible for the drive and the host’s suppression strategies often remain unidentified [11].

In recent decades, research in *Drosophila* has significantly advanced our understanding of sex chromosome drive and its suppression during male gametogenesis. The *Stellate* (*Ste*) locus in *Drosophila melanogaster* is thought to be a relic meiotic drive system, derived from the amplification of the testis-specific β subunit of protein kinase, *CkIIβ2* on the X and Y chromosomes [12-15]. The *suppressor of Stellate* [*Su*(*Ste*)] on the Y chromosome silences X-linked *Ste* copies via post-transcriptional gene silencing, primarily employing the piwi-interacting RNA (piRNA) pathway, which plays a crucial role in defending against transposable elements (TEs) in the animal germline [16,17]. The genetic characterization of *Ste* locus in *D. melanogaster* thus provided the first evidence linking small RNA-mediated silencing of endogenous selfish genes involved in meiotic conflicts between sex chromosomes.

In *Drosophila simulans*, a sibling species of *D. melanogaster*, several X-linked drive systems that are absent in *D. melanogaster* have been identified, highlighting the dynamic evolution of sex chromosome conflicts between closely related species. Interestingly, genetic and molecular characterization of these X-linked drive systems—namely, *Paris* [18], *Durham* [19], and *Winters* [20,21]—have identified the surprising roles of small RNA pathway components in meiotic drive and suppression. In the *Paris* system, a young and rapidly evolving gene called *HP1D2* functions as a meiotic driver to prevent proper Y chromosome segregation. Notably, *HP1D2* is a paralog of *rhino*, a gene involved in activating the transcription of piRNA clusters, which generate piRNA-class small RNAs in *Drosophila* [18,22]. In the *Winters* and *Durham* systems, recent studies have uncovered the crucial roles of the endogenous RNA-interference (RNAi) pathway in defending X-linked meiotic drive genes from the *Distorter on the X* (*Dox*) family drivers [23-25]. Interestingly, evolutionary tracing of the origins of the *Dox* family genes in *D. simulans* revealed their relationship to *Protamine*, a gene involved in sperm chromatin packaging [24,26].

A typical pattern observed in the meiotic drivers of *D. melanogaster* and *D. simulans* is that these genes are often truncated or duplicated versions of otherwise normal genes essential for cellular and developmental functions. Thus, the mechanisms of how meiotic drive genes unleash selfish activities to compromise gametogenesis remain unclear. Nevertheless, studies in the *melanogaster* complex have advanced our understanding of the suppression of sex chromosome meiotic drive. While the primary role of the piRNA pathway is to suppress TE activity in the germline, recent studies in *Drosophila* have uncovered a sexually dimorphic piRNA program with distinct functions during male gametogenesis, including its involvement in suppressing meiotic conflicts [27,28]. Thus, these studies have extended our understanding of piRNA pathway silencing beyond TEs.

In contrast, the RNAi pathway, where small-interfering RNAs (siRNAs) mediate silencing, has been widely employed as a powerful experimental gene silencing tool for the past two decades, though its endogenous utilities have remained mysterious. Like the piRNA pathway, the RNAi pathway is an ancient small RNA-mediated defense mechanism against genome invaders. However, the substrate for the generation of small RNAs and the Argonaute family proteins that siRNAs and piRNAs associate with are distinct between these two pathways (reviewed in [29]). In *D. simulans*, the RNAi pathway was shown to be crucial to controlling X-linked meiotic drivers from the *Dox* family, thereby safeguarding the integrity of male gametogenesis [23-26]. Furthermore, the unsuspected roles of the RNAi pathway in defending intragenomic conflicts may be more widespread than previously thought, as studies in plants have recently demonstrated its crucial role in silencing pollen drive during fertilization [30].

**Figure 1:**
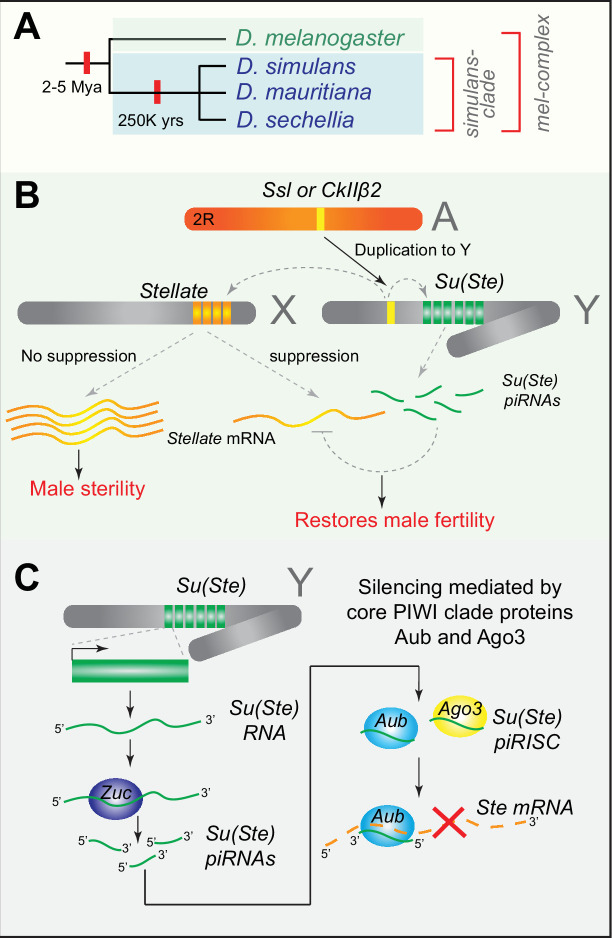
*Stellate* system and piRNA-mediated suppression of sex chromosome meiotic conflicts in *D. melanogaster*. (**A**) Phylogeny of *D. melanogaster* complex. *D. melanogaster* and the *simulans*-clade species diverged ~2–5 million years ago. The three species in the *simulans* clade diverged approximately 250,000 years ago. (**B**) Evolution of *Stellate* (*Ste*) and *Suppressor of Stellate* [*Su*(*Ste*)] in *D. melanogaster*. Autosomal paralogs *Suppressor of Stellate-like* (*Ssl*) or β subunit of protein kinase, *CkIIβ* duplicated to the Y chromosome and further amplified on the X and the Y chromosomes. The X-linked copies have protein-coding potential, whereas the Y-linked *Su(Ste*) copies encode piRNAs. In the presence of *Su(Ste*) piRNAs, active *Ste* copies are silenced, leading to fertile males. However, in the absence of *Su(Ste*) piRNAs, overexpression of X-linked *Ste* copies leads to crystalline aggregates during spermiogenesis, resulting in male sterility. (**C**) Mechanism of piRNA mediated silencing of *Ste* transcripts. The *Su(Ste*) locus encodes non-coding RNAs as piRNA precursors that the nuclease Zuc processes to form piRNAs. These piRNAs are then loaded on to PIWI clade proteins Aub and Ago3 to form the piRNA-induced silencing complex (piRISC). The piRISC scans *Ste* RNAs for degradation via post-transcriptional gene silencing.

This review aims to highlight the emerging and crucial roles of small RNA-mediated suppression of meiotic conflicts during male gametogenesis, focusing on studies from *Drosophila*, notably *D. melanogaster* and closely related *simulans* clade, which consists of three species, namely, *D. simulans*, *D. mauritania, and D. sechellia* (Figure 1A). Selfish genes like meiotic drivers impose a net negative fitness effect on reproductive function. Therefore, the evolution of novel suppression strategies serves to maintain optimal reproductive fitness and inevitably shapes the evolution of germline development and reproduction. As a tangential perspective, the review also aims to shed light on the emerging understanding of how the rapid co-evolutionary dynamics between meiotic drive and suppression affect genome evolution and speciation.

### *Stellate* system and piRNA-mediated suppression of meiotic conflicts

The *Ste* locus and its suppressor, *Su(Ste),* are testis-expressed tandem duplications on the X and Y chromosomes, respectively [31]. *Ste* duplicate copies are related to a testis-specific paralog of β subunit of protein kinase, *CkIIβ* [32], and the suppression of X-linked *Ste* by Y-linked *Su(Ste*) is found only in *D. melanogaster,* although homologous sequences are found on the X and Y in other *simulans*-clade species [33,34]. This indicates that the *Ste* and *Su(Ste*) silencing via piRNAs is a unique adaptation in *D. melanogaster* to mitigate sex chromosome conflict. The X- and Y-linked copies emerged from the duplication of *suppressor of Ste-like* (*Ssl*)—an autosomal paralog of *CkIIβ2* that first duplicated to the Y-chromosome, followed by amplification on the X and Y chromosomes [14] (Figure 1B). Deletions encompassing the *Su*(*Ste*) locus on the Y chromosome result in the derepression of X-linked *Ste* copies, leading to meiotic defects during male gametogenesis [31,35].

Genetic studies of chromosome segregation in *Su(Ste*) mutant males found that both sex chromosomes and autosomes undergo non-disjunction (i.e. fail to segregate properly during meiosis), revealing a phenotype consistent with meiotic drive [35]. In addition, the derepression of the *Ste* locus on the X-chromosome leads to needle-shaped crystalline aggregates in the nuclei and cytoplasm of primary spermatocytes. Critically, the severity of the phenotype depended on the strength of allelic states and copy number of the *Ste* locus [32]. Combined with the male sterility phenotype observed in *Su(Ste*) mutant males, these observations led to the notion that *Ste* is a remnant of intragenomic conflicts between the sex chromosomes in *D. melanogaster* [12,13].

Interestingly, the suppressor, *Su*(*Ste*) on the Y-chromosome lacks protein-coding potential and is highly divergent at the sequence level compared with the X-linked *Ste* copies [36]. Due to the presence of multiple copies on both genomic strands, *Su(Ste*) produces sense and antisense RNA complementary to *Ste*, which are processed into small RNAs and mediate the silencing of *Ste* genes on the X chromosome (Figure 1C) [16,37]. Indeed, pioneering studies on the *Stellate* system in *D. melanogaster* led to identifying a germline-specific small RNA pathway—now widely known as the piRNA pathway [16,36,38]. Additional studies have since revealed the essential role of piRNA pathway in suppressing TEs in the germ line, a highly conserved function across animals [39]. piRNA-mediated target silencing occurs through the assembly of an RNA-induced silencing complex (RISC), in which the Argonaute family proteins—Aub or Ago3—bind to piRNAs and silence complementary target RNAs via post-transcriptional silencing (Figure 1C). In contrast, Piwi, another Argonaute member in the piRNA pathway, is involved in co-transcriptional silencing in the nucleus [40].

In addition to understanding sex chromosome meiotic conflicts, the *Stellate* system in *Drosophila* has also served as an excellent model to investigate the sex-specific mechanisms of piRNA-mediated post-transcriptional silencing. For instance, in *Drosophila*, maternally derived piRNAs have an epigenetic role in triggering piRNA production for effective TE silencing in the progeny [41]. If so, how Y-linked piRNA production from the *Su(Ste*) could be initiated remained an unsolved question. Interestingly, a recent study revealed that *Su(Ste*) copies on the Y chromosome, which have a *1360*/*Hoppel* TE insertion at the 5′ end adjacent to *Su(Ste*), act as a trigger. Here, maternal *1360*/*Hoppel* piRNAs guide the production of *Su(Ste*) piRNAs, leading to *Ste* silencing in males [42]. These recent studies on the *Stellate* system highlight the highly adaptable nature of piRNA-mediated silencing in mitigating active and emerging meiotic conflicts.

While extensive research has uncovered the intricacies of the piRNA pathway’s role in TE silencing, the emerging mechanics of relaxed piRNA-targeting rules [43] suggests that piRNA-mediated silencing is a highly adaptable system capable of targeting rapidly diverging sequences—an evolutionary hallmark of genes like *Ste* involved in intragenomic conflicts. However, how often the piRNA pathway gets recruited to silence endogenous selfish genes like *Ste* and, in general, sex chromosome conflicts remains unclear. New studies examining the sex-specific piRNA program in the *Drosophila* male germline have uncovered piRNA targeting of novel endogenous genes such as *nod* and *paics,* and *petrel,* which targets the endogenous gene *pirate*, appears to be multi-copy genes on the sex chromosomes like *Ste* [27]. However, it remains to be determined whether these novel endogenous piRNA targets play a role in meiotic conflicts. Nevertheless, the role of piRNA pathway in defending endogenous selfish genes has expanded our understanding of piRNA targeting beyond TEs, highlighting its crucial function in resolving intragenomic conflicts between the sex chromosomes.

### *Dox* system and RNAi suppression of sex chromosome drive

**Figure 2:**
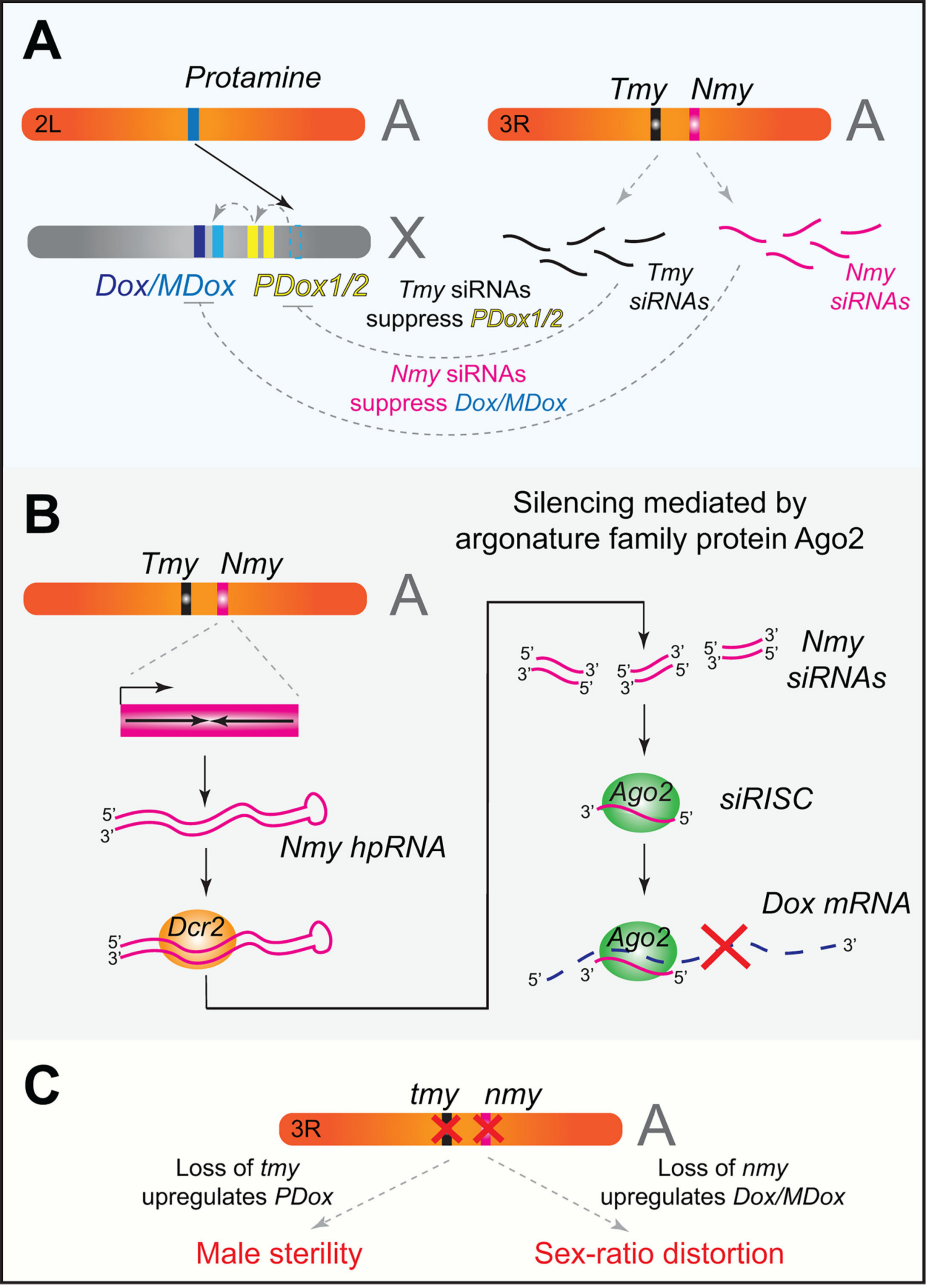
*Dox* system and RNAi-mediated suppression of sex chromosome drive in *D. simulans*. (**A**) Autosomal *Protamine* gene duplicated to the X-chromosome to spawn the *Dox* family genes in *simulans* clade. The dotted blue box on the X-chromosome represents the *Proto-Dox* (or *Ur-Dox*) inferred from evolutionary analyses but absent in the contemporary *w[XD1] D. simulans* strain. Diversification of ancestral *Ur-Dox* led to the emergence of novel members of the *Dox* family genes, namely, *ParaDox* (*PDox1* and *PDox2*), *Mother of Dox* (*MDox*), and the *Distorter on the X* (*Dox*). The expansion of *Dox* family genes led to the evolution of novel hairpin RNA (hpRNA) suppressors, *Too much yin* (*Tmy*) and *Not much yang* (*Nmy*) on the autosome. *Nmy* and *Tmy* encode endo-siRNA-class small RNAs with target specificity to *Dox*/*MDox* and *PDox* genes, respectively. (**B**) *Nm*y hpRNA as an example for siRNA-mediated targeting of *Dox*. The *Nmy* locus encodes an inverted-repeat, which forms double-stranded RNA (dsRNA) and is a substrate for the Dcr2 endonuclease processing. The dsRNA is processed into predominantly 21 nucleotide small RNAs that are loaded onto the effector argonature protein Ago2 to form RISC, which scans *Dox*/*MDox* mRNA for degradation based on complementarity. (**C**) Distinct spermiogenic phenotypes in *nmy* and *tmy* mutants. In a *nmy* mutant, the loss of *Nmy* siRNAs results in the upregulation of *Dox* and *MDox* genes, leading to a sex-ratio distortion phenotype. On the other hand, in a *tmy* mutant, the loss of *Tmy* siRNAs results in a male sterility phenotype.

The hybrid male sterility resulting from inter-species hybridization among the three species in the *simulans* clade (Figure 1A) makes it an excellent model for studying the genetics of speciation [44]. These studies led to the original observations of sex-ratio distortion and the discovery of sex chromosome drive in *D. simulans*. In the *Durham* drive system, a dominant suppressor called *Too Much Yin* (*Tmy*) was found to inhibit X-linked drive, although the specific meiotic driver(s) on the X chromosome has not been identified [19]. Similarly, for the *Winters* system, an inverted repeat suppressor, *Not Much Yang* (*Nmy*) was found to suppress an X-linked driver called *Dox*, suggesting the likely involvement of homology-dependent silencing in controlling sex chromosome drive [20,21]. Homology analyses could not identify the orthologs of *Dox* in *D. melanogaster*, suggesting that *Dox* might be a novel gene in *D. simulans*. Interestingly, the discovery of a paralog of *Dox*, known as *Mother of Dox* (*MDox*), led to the notion that the meiotic drive gene *Dox* evolved from its ancestral gene, *MDox* [21]. Further investigations clarified that the *Nmy* locus encodes hairpin RNA (hpRNA) class endo-siRNAs [45], which are processed into predominantly 21 nucleotide siRNAs by the RNAi machinery that targets *Dox* and *MDox in trans* for silencing (Figure 2A) [23]. The cellular RNAi toolkit encompasses the ribonuclease Dcr2, which generates small RNAs from double-stranded RNA substrates that are then loaded onto the Argonaute family member Ago2 for RISC activity (Figure 2B) [46]. Interestingly, in *nmy* mutants, both *Dox* and *MDox* are derepressed [23], resulting in a sex-ratio distortion phenotype. This suggested that *MDox* may also function as a meiotic driver, although the individual contributions of *Dox* and *MDox* to the drive phenotype are yet to be determined. Furthermore, genomic analyses revealed that the *Tmy* locus encodes a novel hpRNA and displays significant homology to *Nmy*, suggesting that the *Winters* and *Durham* drive systems are evolutionarily inter-connected. Indeed, improved genome assemblies in *D. simulans* [47] identified two additional genes related to *Dox* and *MDox*, namely, *ParaDox* (*PDox1* and *PDox2*), on the X-chromosome that are highly homologous to *Tmy*, suggesting that the different *Dox* family genes and their concomitant hpRNA suppressors may have originated during repeated bouts of meiotic drive and suppression (Figure 2A) [24,26].

Since the closest sister species, *D. melanogaster*, lacks any of these hpRNA suppressors, the origins of the *Dox* family genes remained unclear for over a decade. Detailed evolutionary tracing led to the identification that the meiotic driver *Dox* emerged via a chimeric fusion by acquiring segments from multiple *D. melanogaster* genes with its open-reading frame originating from *Protamine* [24,26]. Protamines are sperm nuclear basic proteins (SNBPs) involved in chromatin compaction. Many SNBP factors are essential for male fertility and are known to exhibit signatures of rapid evolution [48,49]. *Dox* family genes encode a high mobility group (HMG)-box domain that is predicted to bind DNA—a domain typically found in many transcription factors and, more importantly, in Protamine-like SNBPs [50]. However, the HMG-box domain in *Dox* appears to be highly divergent from the *Protamine*, suggesting rapid diversification of *Dox* family genes, likely due to its role in intragenomic conflicts.

Mechanistically, how *Protamine*-like *Dox* family genes derail male gametogenesis is yet to be understood; however, the chromatin association of PDox2 during telophase of meiosis I and II lends support to their potential roles in the misregulation of the chromatin landscape during spermiogenesis [25]. In contrast, Dox sub-cellular localization was found to be primarily cytoplasmic during meiosis and was not detected during postmeiotic stages, while canonical protamines exhibit exclusive nuclear localization during postmeiotic stages [25]. In line with these observations, the loss of *tmy* and *nmy* hpRNA has marked differences in spermiogenic phenotypes exhibiting male sterility and sex-ratio distortion, respectively, due to derepression of distinct *Dox* family genes (Figure 2C). These findings suggest that although *Dox* factors have originated from protamines, their subcellular localization paints a complex picture consistent with likely neo-functionalization. Therefore, whether the *Dox* family members may function as protamine mimics or have acquired other novel function(s) is yet to be determined.

Furthermore, comparative evolutionary analyses of *Dox* family genes have revealed that the drive and suppression system originated in the *simulans*-clade ancestor [24]. Interestingly, the *Dox* family genes have undergone massive amplification on the X chromosomes in *D. mauritiana* and *D. sechellia*, with 22 additional copies identified across both genomes. In most instances of these amplified copies, the *Dox* family duplicates lack synteny and orthology, indicating that multiple independent insertions have occurred across all three *simulans*-clade species. Detailed evolutionary tracing on the origins of *Dox* family genes has also revealed the association of *Dox* genes with a family of satellite repeats called 359-satellite, which led to the amplification of these genes on the X-chromosome [51]. Crucially, the expanding cohort of *Dox* genes has also birthed novel *Tmy*-like hpRNAs, namely, *Tmy2* in *D. mauritiana* and *m-Tmy-C* in *D. sechellia*.

The rapid expansion of a family of meiotic drivers and their hpRNA suppressors is a testament to an ongoing genetic arms race in the *simulans* clade. Indeed, CRISPR-Cas9 mutagenesis of the core RNAi factor *Dcr2* in *D. simulans* uncovered many *de novo* hpRNAs like *Nmy* and *Tmy* with predominantly X-linked targets. These novel hpRNAs and their targets are absent in *D. melanogaster*, indicating RNAi pathway’s unexpected broader role in mitigating other likely meiotic conflicts beyond the *Dox* family genes in *D. simulans* [52]. Thus, compromising the RNAi machinery in *D. simulans* unveiled novel, dynamically evolving hpRNA-target networks, highlighting how germline genetic conflicts can invariably alter genome evolution among closely related and incipient *Drosophila* species.

## Summary and outlook

Past and recent research on sex chromosome drive in *Drosophila* has uncovered common themes, particularly from the detailed study of a few examples in *D. melanogaster* and *D. simulans*. First, genes identified to cause sex chromosome drive in these species tend to be duplicates of ordinary genes with a history of amplification on the sex chromosomes [24,26,36]. Interestingly, another well-known example of an autosomal meiotic driver in the segregation distortion (*SD*) system in *D. melanogaster* is a Ran GTPase activating protein (*RanGAP*) [53,54]. RanGAP is also seemingly a normal gene, which acts as a GTPase activator for the nuclear Ras-related regulatory protein *Ran* [55]. Interestingly, *SD-*mediated meiotic drive results in the compromised histone-to-protamine transition [56], and knockdown of protamines in *D. melanogaster* was shown to induce distortion in the *SD* background [57]. These studies highlight a recurring theme in connecting protamines to meiotic drive. On the connected theme of protamines, Y-linked protamine-like *Mst77Y*, which are paralogs of autosomal *Mst77F* required for histone-to-protamine transition, were shown to compromise the compaction of the X-chromosome when misexpressed [58]. Although the effect was modest on sex-ratio distortion, the regulation of *Mst77Y* is mediated by a nucleolar protein called Modulo*,* highlighting other potential means to resolve meiotic conflicts that do not involve small RNA pathways.

The second common trend observed is the association of meiotic drive genes with repetitive DNA, either as a template for amplification, as in the case of the Dox system, or for regulation, as exemplified by the *1360*/*Hoppel* repeats in *Su(Ste*) system. Interestingly, the strength of meiotic drive in the *SD* system is also associated with *Responder* (*Rsp*) satellite repeats, highlighting genomic repeats as significant players in the emergence and maintenance of meiotic drive. Therefore, one of the future challenges is to uncover how (and when) duplicate or ampliconic genes acquire selfish properties and what roles genomic repeats may play in the evolution of meiotic drive.

Studies in *D. melanogaster* and *D. simulans* have also uncovered the crucial importance of small RNA pathways in defending against meiotic drive. Both piRNA and siRNA pathways are deeply conserved in evolution [39], and the recruitment of piRNA pathway to silence *Stellate* genes in *D. melanogaster* and the RNAi machinery in counteracting *Dox*-mediated sex chromosome drive in *D. simulans* attest to the evolutionary plasticity in the use of different small RNA pathways for silencing intragenomic conflicts. These studies also highlight how small RNA-based silencing strategies can be a quick and potent response against emerging meiotic conflicts and may explain their deep conservation across evolution and sexually dimorphic functions, as revealed by a recent study [59].

Indeed, the remarkable flexibility in recruiting different small RNA pathways to combat potential meiotic conflicts was recently shown for the endogenous gene, *pirate,* in *D. melanogaster*, targeted by Y-linked piRNAs in the male germline. Interestingly, in the closest sister species *D. mauritiana*, *pirate* is targeted by siRNAs instead of piRNAs, highlighting the variability in the use of different small RNA pathways to target the same gene [27]. Thus, in the evolution’s toolkit to defend against emerging meiotic conflicts, these small RNA pathways can be viewed as a ‘Swiss Army Knife’ for effectively responding to intragenomic conflicts. Therefore, the observations from *Drosophila* studies raise the question of how commonly small RNA pathways combat sex chromosome conflicts and meiotic drive during male gametogenesis. With high-quality genome assemblies becoming available in many non-model *Drosophila* species [60], small RNA profiling from the male germline in multiple *Drosophila* species and other dipteran insects may help uncover the utility and variation among these pathways in targeting endogenous selfish genes.

Finally, studies on sex chromosome drive in *Drosophila* have uncovered surprisingly rapid evolutionary dynamics between selfish genes and their small RNA suppressors. Thus, it is becoming increasingly apparent that these male meiotic conflicts can potently shape the evolutionary trajectories of genes and genomes. For instance, the *Dox* family meiotic drive genes and their concomitant suppressors in *D. simulans* are not only lineage-specific but also exhibit remarkable variation in terms of expansion in copy numbers of *Dox* factors and the evolution of multiple *Tmy*-like suppressors in the *simulans* clade that emerged from repeated cycles of meiotic drive and suppression [24,26].

One of the consequences of rapid evolution from intragenomic conflicts is that the drive and suppression can compromise both reproductive fitness in the species harboring these drive elements and during hybridization between closely related species. Indeed, in a few examples from non-model *Drosophila* species, sex chromosome drive is either directly associated with hybrid sterility [61] or the sex chromosome meiotic driver is causal for reproductive isolation [62,63]. However, how frequently meiotic drive acts as an evolutionary force to propel reproductive isolation and speciation remains to be investigated. As sex chromosome drive is known to occur in many *Drosophila* species and dipteran insects [64,65], future studies on small RNA profiling, in conjunction with high-quality genome assemblies from these species, could answer the breadth of the usage of RNAi or piRNA pathways in mitigating meiotic drive. Recently, the expanding scope of genome sequencing and small RNA profiling from insects such as *Bombyx mori* and *Plutella xylostella* have linked a role for piRNAs in reproduction and the sex determination cascade [66,67]; however, it remains to be seen how frequently small RNA pathways are involved in suppressing meiotic sex chromosome conflicts.

**Perspectives**

- Selfish genes such as meiotic drivers violate Mendelian segregation, and their selfish activities can compromise germline development and reproduction. Negative fitness effects arising from meiotic drive lead to the evolution of suppressors to restore the *status quo*. The rapid evolutionary dynamics of meiotic drive and suppression can, therefore, be a potent force in shaping the evolution of germline processes.
- Studies in *Drosophila* have revealed the crucial importance of small RNA pathways, such as the piRNA and RNAi pathways, in controlling the selfish activities of meiotic drive genes during male gametogenesis. The examples of *Drosophila* meiotic drive genes that mediate sex chromosome drive are duplicates or diverged copies of otherwise normal genes, indicating that seemingly ordinary genes can acquire selfish properties in the meiotic landscape.
- The molecular identities of meiotic drivers known so far in *Drosophila* provide clues to examining their potential mechanisms of action in derailing male gametogenesis. In addition, the findings that small RNAs are at the crux of meiotic drive suppression can lead to future studies examining the extent and usage of small RNA pathways to combat drive in other non-model *Drosophila* and dipteran insect species.

## Abbreviations

Dox: Distorter on the X
HMG: high mobility group
MDox: Mother of Dox
Nmy: Not Much Yang
RNAi: RNA-interference
RanGAP: Ran GTPase activating protein
Rsp: Responder
SD: segregation distortion
SNBPs: sperm nuclear basic proteins
Ste: Stellate
Su(Ste): Suppressor of Stellate
TEs: transposable elements
Tmy: Too Much Yin
hpRNA: hairpin RNA
piRNA: piwi-interacting RNA
siRNAs: small-interfering RNAs
